# COVID-19 Vaccination and Predictive Factors in Immigrants to Europe: A Systematic Review and Meta-Analysis

**DOI:** 10.3390/vaccines12040350

**Published:** 2024-03-25

**Authors:** Emanuela Gualdi-Russo, Luciana Zaccagni

**Affiliations:** 1 Department of Neuroscience and Rehabilitation, Faculty of Medicine, Pharmacy and Prevention, University of Ferrara, 44121 Ferrara, Italy; luciana.zaccagni@unife.it; 2 Center for Exercise Science and Sports, University of Ferrara, 44123 Ferrara, Italy

**Keywords:** COVID-19, vaccine uptake, immigrants, Europe, barriers, vaccination intention

## Abstract

Vaccination plays a pivotal role in the control of infectious disease outbreaks. Hesitancy/refusal of the vaccine by immigrants poses a serious threat to their and society’s health. We reviewed studies regarding COVID-19 vaccine uptake in Europe by first-generation immigrants. A systematic review (PROSPERO: CRD42023432142), conducted until 31 October 2023 using Web of Science, PubMed, and Scopus, identified 295 potential articles. Of these, 16 conducted on 2,009,820 immigrants in nine European countries met the eligibility criteria. Most studies were of medium/high quality according to the Newcastle–Ottawa Scale adapted for observational studies. Factors that affected the uptake or hesitancy/refusal to vaccinate, with particular regard to gender, age, and country of origin, were examined. The meta-analysis of eight studies revealed that the pooled estimated prevalence of COVID-19 vaccine uptake in first-generation immigrants was 71.3% (95% CI: 70.0–72.5%), corresponding to 13.3% less than the host country population (95% CI: 10.2–16.4%). Limitations of included studies and this review were deeply discussed, highlighting the need for further research on the effect of acculturation on second-generation immigrants. European governments need to ensure equal availability of COVID-19 and other health-saving vaccines to all immigrants in the future by overcoming cultural barriers, building trust in institutions, and improving communication.

## 1. Introduction

The first cases of pneumonia of unknown etiology were detected in Wuhan (China) in 2019, and their cause was later identified to be a new coronavirus (SARS-CoV-2) in January 2020. Meanwhile, starting with the first isolated cases discovered in France and Italy as early as January 2020, all European states have been heavily affected by COVID-19. The 2019 global epidemic of coronavirus was declared by the WHO in March 2020 [1]. More than three years after its outbreak (5 May 2023), the WHO has declared the end of the global COVID-19 epidemic.

To avoid the consequences of the disease, attempts have been made to vaccinate as many people as possible throughout the world including Europe [2] to particularly reduce severe forms, hospitalizations, and deaths. Several vaccines have been developed for this purpose, including mRNA-based vaccines (Moderna and Pfizer), inactivated whole-virus vaccines (Sinovac and Sinopharm), or recombinant vaccines (e.g., Oxford–AstraZeneca and Johnson & Johnson) [3]. The first vaccinations against COVID-19 were administered in Europe one year after its emergence, namely on 27 December 2020, symbolically designated as Vaccine Day [4]. Governments have strongly supported COVID-19 massive vaccination programs, but the outcome has been uncertain among some population groups, partly due to several vaccine side effects, including local and systemic. Continuous monitoring of vaccine safety has been carried out by the European Medicines Agency [5], which has thus been able to show that the vast majority of reported side effects have been mild to moderate, while serious side effects have been very rare.

The International Organization for Migration [6] solicited governments to include immigrants, including undocumented immigrants, in vaccination campaigns: the safety of all is closely linked to the protection from health risks that is provided to the most vulnerable because of their non-stable or transient status.

Of the 103 million people who were refugees in various countries around the world in 2022 according to the United Nations High Commissioner for Refugees (UNHCR), there were 23.8 million non-EU citizens in Europe in the same year corresponding to 5.3% of the total European population with 140 different countries of origin for asylum seekers [7]. This population was at a high risk of virus transmission due to various causes, such as overcrowding, lack of access to clean water and sanitation, and inadequate medical care. Even so, several factors may have contributed to poor vaccination of some immigrant groups, or they may have faced barriers to accessing [8]. As a result of existing cultural barriers, especially in the access to healthcare services, a lower rate of COVID-19 vaccination was reported, for example, in foreign populations than native ones in Italy, one of the most affected Western countries [9].

A recent review [10] analyzed the reason for the general under-immunization of immigrants living in one of 30 countries of the European Union or European Economic Area (in addition to Switzerland and the UK) according to the literature published from 1 January 2000 to 14 September 2021 and indicated that several barriers to vaccinations exist for immigrants from language and legal barriers to service barriers due to lack of service guidelines. Individuals of all ages and all types of vaccines (including those for COVID-19) were considered in the cited review.

Unlike previous reviews, this one focuses on vaccination against COVID-19 in immigrants located in one of the 50 countries on the European continent. The situation in which the vaccination campaign took place differed strongly from the others because of the mental condition of anxiety and alarm generated in populations by the ongoing epidemic accentuated by the periods of lockdown and social isolation that characterized it. This is, to our knowledge, the first study conducted at the end of the pandemic that seeks to understand, through a systematic review of the existing literature, possible differences in immigrants’ decision to vaccinate against COVID-19 in Europe. Assessing the hesitancy of immigrants, defined as vaccination refusal or delayed acceptance [11], is therefore of great interest by examining its trend with ethnic background and the various possible factors that motivated it. For this purpose, this review aims to systematically examine the studies found in the literature related to the epidemic period to assess vaccination aptitudes for COVID-19 demonstrated by first-generation immigrants in European countries in relation, when possible, to age, sex, country of origin, education level, working status, and health condition. Understanding these issues is essential to properly target health promotion interventions to improve vaccination uptake now and soon when needed.

## 2. Materials and Methods

We performed this systematic review according to PRISMA guidelines (Preferred Reporting Items for Systematic Reviews and Meta-Analyses) [12] and reported the PRISMA 2020 for abstracts checklist in Table S1 and the PRISMA 2020 checklist in Table S2 (Supplementary Materials). The protocol registration of this review is PROSPERO: CRD42023432142 (17 July 2023) available from https://www.crd.york.ac.uk/prospero/display_record.php?ID=CRD42023432142.

### 2.1. Search Strategy and Criteria of Selection

PubMed, Web of Sciences (WoS), and Scopus databases were systematically examined in the search for articles published during the COVID-19 epidemic until 31 October 2023. The search terms in the literature review were reported in the following combination: (COVID* OR SARS-CoV-2 OR coronavirus) AND ((vaccin* OR immunization) AND (hesitancy OR refusal OR coverage OR acceptance OR uptake OR willingness OR intention OR barrier*)) AND (immigrants OR migrants OR refugees OR asylum OR foreign-born) AND (Europe OR Austria OR Belgium OR Bulgaria OR Cyprus OR Croatia OR “Czech Republic” OR Denmark OR Estonia OR Finland OR France OR Germany OR Greece OR Hungary OR Iceland OR Ireland OR Italy OR Latvia OR Liechtenstein OR Lithuania OR Luxembourg OR Malta OR Netherlands OR Norway OR Poland OR Portugal OR Romania OR Slovakia OR Slovenia OR Spain OR Sweden OR Switzerland OR “United Kingdom” OR Albania OR Belarus OR Ukraine OR Russia OR Moldavia OR “North Macedonia” OR “Bosnia and Herzegovina” OR Serbia OR Montenegro OR Andorra OR Monaco OR Armenia OR Turkey OR “San Marino” OR Kazakhstan OR Georgia OR Azerbaijan OR “Vatican City”).

An independent review of the articles based first on the titles and abstracts and then on the whole text was carried out by both authors (E.G.-R., L.Z.). In cases of dispute over the eligibility of an article, consensus was reached through further analysis and discussion among the authors. In the end, the search for additional articles was completed by analyzing the references listed in the selected articles.

Following the PECO framework [13], we considered the following eligibility criteria: population, exposure, comparator, and outcomes. The studied populations were regular or undocumented immigrants of the first generation, including refugees and asylum seekers, who frequently encounter social, cultural, administrative, financial, legal, and linguistic barriers to healthcare system access [1]. They were to be located in a European country during the pandemic and be of an age compatible with vaccination. The exposure considered was to the pandemic COVID-19. The comparisons considered were with populations other than immigrants, i.e., the autochthonous populations of the European countries under consideration. The primary outcomes referred to data on immigrants’ access to or hesitancy/refusal of COVID-19 vaccines; secondary outcomes concerned main factors of hesitancy/refusal or barriers in vaccine uptake. Moreover, we considered only peer-reviewed articles with full-text availability. In addition to the articles in English, articles in three other European languages well known to the authors (Italian, Spanish, and French) were also considered to make this review as representative as possible of the published findings on the epidemic that affected Europe.

Exclusion criteria were qualitative studies, studies of clinical populations, ethnic minorities of people born in the host country, persons under the age of 16 years or belonging to particular age groups of immigrants (e.g., only adolescents or only the elderly). Population studies that did not report results for immigrants were excluded, as were those that returned overall population results that could not be disaggregated. Articles aimed at proposing facilitation in vaccine access policies through the development of new tools or apps were also excluded because they did not address our outcome of interest. Studies related to immigrants’ access to vaccines other than the COVID-19 vaccine were further excluded. Finally, we excluded literature reviews, abstracts, books (or book chapters), editorials and commentaries, reports, case studies, protocol studies, letters, and conference papers.

The authors extracted independently the following data from each study, where available: i. Study characteristics and methods (authors’ name and year of publication, study design, sampling period, data collection method); ii. Information on participants (European host country, country of origin, sample characteristics—size, sex, ethnicity, education, income, comorbidities); iii. Outcomes (vaccination prevalence for COVID-19, hesitation/refusal to vaccinate, and main factors involved). A qualitative summary of the retrieved data has been shown in table form, reporting the studies in alphabetical order.

### 2.2. Quality Assessment

Quality assessment of the included studies was performed independently by the two reviewers using the adapted Newcastle–Ottawa Scale (NOS) for observational studies [13]. Up to 16 scores were assigned to each study based on component ratings (clarity of objectives based on 1 issue, sample selection based on 4 issues, comparability based on 2 issues, and outcome based on 2 issues). The ascertainment of exposure, included in the sample selection, was estimated regarding the use of official immunization registries and/or validated questionnaires. Following Hillen et al. [14], the studies with scores 13 to 16 were considered high quality (scores > 75%), 9 to 12 moderate quality (scores > 50%), and 1 to 8 low quality (scores ≤ 50%) and higher risk of bias. Anyway, for the sake of transparency, we decided not to exclude any study based on the quality assessment. Any disagreements between the two reviewers were resolved by careful review and discussion.

### 2.3. Data Analysis

We meta-analyzed the data regarding the prevalence of COVID-19 vaccine uptake using MedCalc^®^ Statistical Software version 22.017 (MedCalc Software Ltd., Ostend, Belgium; https://www.medcalc.org; 10 January 2024). We reported the pooled prevalence of COVID-19 vaccine uptake and its 95% CI using a random-effects model. Heterogeneity was assessed using the I^2^ statistic, which indicates the proportion of total variation among the effect estimates attributed to heterogeneity rather than sampling error. A value of I^2^ equal to 0% indicates that there is no heterogeneity, while higher values indicate heterogeneity growing from low (25%), to moderate (50%) or high (75%) [15]. A forest plot was used to display the results of the meta-analysis. Egger’s test and funnel plot were used to assess publication bias among the included studies. A *p*-value > 0.05 indicates the absence of significant evidence of publication bias.

## 3. Results

### 3.1. Screening and Selection Process

**Figure 1:**
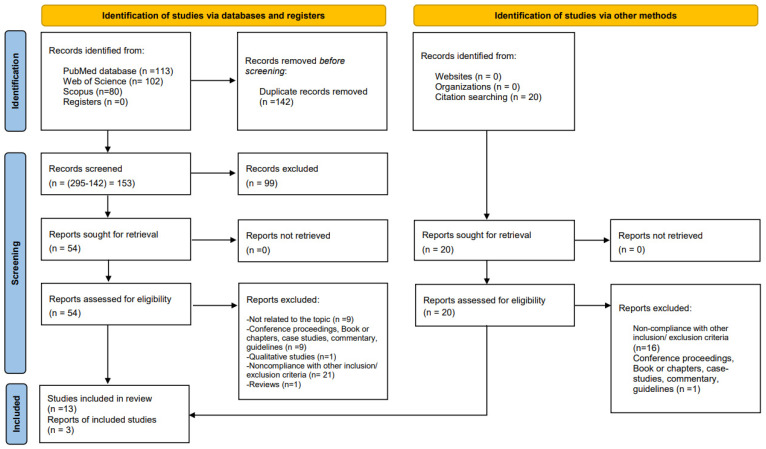
Flowchart of the selection process of the study (PRISMA 2020 flow diagram) [12].

We conducted the initial search in three databases with the retrieval of 295 records: PubMed (n = 113); Scopus (n = 80); and WoS (n = 102). We then eliminated 142 duplicates and proceeded to screen the rest of the papers based on title and abstract. After removing 99 papers from the remaining 153 given their titles and/or abstracts, we examined the full text of the 54 papers that were potentially electable. As a result of the inclusion and exclusion criteria, 13 studies were selected for final analysis and, after reading their references, three additional studies were added. Therefore, 16 studies were included in the systematic review, and the two meta-analyses conducted considered 8 and 6 studies, respectively, among those selected. The PRISMA flowchart shows the selection process in Figure 1.

### 3.2. Overview of the Included Studies

We included in this review sixteen studies with a total of 2,009,820 immigrants examined in nine European nations (Table 1).

Specifically, five of these were carried out in Norway, two each in France, Germany, and Italy, one each in Finland, Denmark, Sweden, and Turkey, and one multicentric study in Switzerland, France, and Italy. Three studies (19%) were published in 2021 [23,25,29], four (25%) in 2023 [16,18,21,30], and the remaining nine studies (56%) in 2022 [17,19,20,21,22,24,26,27,28]. The earliest data collection was compiled in the period May 2020–June 2020 in Longchamps’ study [25], which investigated the hesitancy to vaccinate, as the COVID-19 vaccine was not yet available; the latest one was compiled on COVID-19 vaccination status in February, March, and May 2022 by Aysit et al. [16].

Immigrant samples consisted of participants of both sexes in all studies. Sample sizes ranged from 204 participants (a convenience sample of immigrants in Germany) [20] in the smallest to over five million (individual-based data from nationwide Danish registries) [21] in the largest.

Six studies had less than 1000 participants, 5 between 1000 and 80,000, and five had more than 1 million participants. Seven studies were conducted only on immigrants; the remainder also involved participants from the host country. Considering only the immigrant sample, the smallest sample size was 150 (a convenience sample of Polish immigrants in Norway recruited via Facebook) and the largest was 709,030 (the immigrants of Western and non-Western descent in Denmark on Danish registries). Seven studies had less than 1000 immigrants, three studies between 1000 and 5000 immigrants, and six had more than 10,000 and less than 709,030. The studies carried out on the largest samples were generally designed as register-based cohort studies with the data obtained from regional or national registries; the other studies were cross-sectional designed based upon data collected by web-based surveys with computer-assisted web interviews (CAWI) or computer-assisted telephone interviews (CATI). Only in one study [25], the participants (residents of French homeless shelters) were interviewed in person or by telephone, while in the multicentric study [28] and in the study on Syrians under “Temporary Protection” in Istanbul [16], the questionnaire was available at health facilities. The participants of the study of Holz et al. [22] were randomly drawn from the database of a third-party online access panel provider and incentivized by earning 50 Euro cents per 10-minute interview time. Concerning the country of origin of immigrants in European countries, the Italian studies [27,29] subdivided the sample into Italians or foreigners according to their citizenship; the others considered immigrants as born abroad of foreign-born parents. Only Kraft et al. [23] considered immigrants separately by country of origin; Lajunen and Wrobel [24] and Aysit et al. [16] considered immigrants from only one country (Poland and Syria, respectively); the other studies subdivided immigrants from countries grouped by regions or by language spoken.

Ten studies aimed at vaccine uptake in immigrants (and in the host population, if investigated) [16,17,18,19,20,21,23,27,29,31], three studies aimed at the vaccine hesitancy [25,28,30], one study the attitude to vaccinate [24], one other [22] the vaccine intention, and the last remaining [26] the information access to preventive measures against COVID-19.

### 3.3. Quality Assessment

We evaluated the quality of the 16 selected studies using NOS with scores ranging from 6 to 15. Eleven articles (accounting for 68.8%) were of moderate or high quality (low risk of bias), and the remaining five articles (accounting for 31.3%) were of low quality (high risk of bias). Examining in detail, more than half of the articles (56.3%) stated the aims accurately in light of the available literature. In sample selection, just under half of the articles (44%) used a representative sample of the average in the target population (all subjects or random sampling), the sample was of justified and satisfactory size, with a response rate >70%, and with a validated measurement tool for ascertaining exposure (Table 2).

### 3.4. Meta-Analyses

#### 3.4.1. The Pooled Prevalence of Vaccination among Immigrant Groups

In this elaboration, we did not consider the sample of Syrian immigrants from Turkey [16], both because of the sex imbalance (81% women) and the late period of data collection (referring to only 2022) compared to the other studies. Through the meta-analysis, we found a high heterogeneity across the studies (I^2^: 99.60%, *p* < 0.001), and we estimated a pooled uptake prevalence of the COVID-19 vaccine of 71.3% (9 samples, 95% CI: 70.02–72.52%) among a cumulative sample size of 1,960,724 immigrants in European countries. The proportion of vaccine uptake in the immigrant samples varies from a minimum of 59.1% in the racially minoritized subsample of immigrants in France [17] to a maximum of 79.9% in two samples of immigrants in Norway [19] and Germany [20]. We have shown in Table 3 the forest plot related to the pooled vaccine uptake prevalence in immigrants.

**Figure 2:**
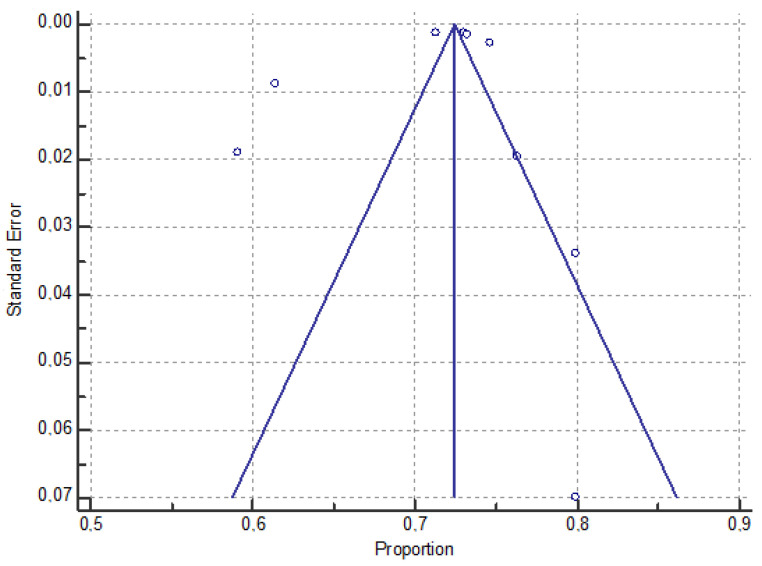
The funnel plot assessing publication bias among selected studies showing prevalence estimates of COVID-19 vaccine uptake among immigrant groups.

No evidence of publication bias was observed by visual inspection of the funnel plot (Figure 2) or by using Egger’s test (*p* = 0.73).

#### 3.4.2. The Pooled Prevalence of Vaccination among Populations with and without an Immigrant Background

**Figure 3:**
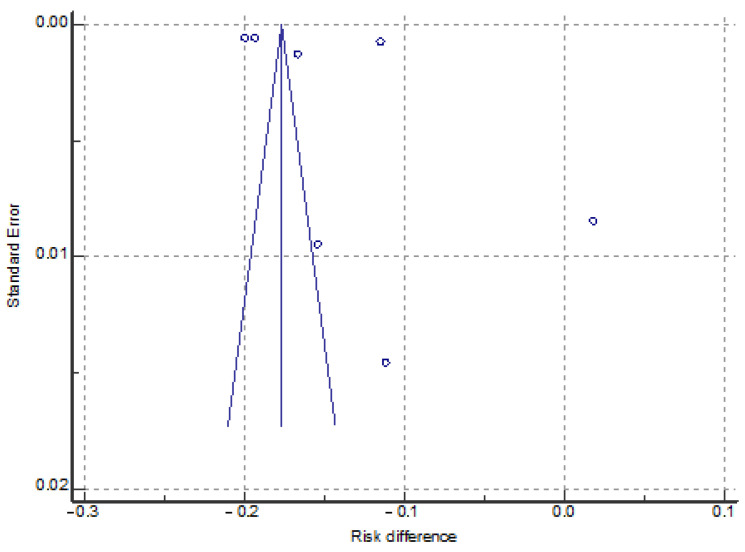
The funnel plot for assessment of publication bias among selected studies reporting immigrant and native populations.

Six studies reported the data of vaccination uptake in seven immigrant groups (total: 1,947,297) and the host populations (total: 11,166,747). In all studies, immigrants reported a lower prevalence of vaccination than the host population, except the sample of non-racially minoritized immigrants in Bajos et al.’s study [17]. The difference in vaccination uptake ranges from a disadvantage for immigrants in Norway of 20.0% in the study of Kraft [23] to an advantage of 1.8% in the immigrants in France in Bajos et al.’s study [17]. Data analysis using the random-effects model revealed that the global difference prevalence was 13.3% (95% CI: 16.4–10.2%). Heterogeneity across the studies was high, as indicated by the I^2^ value (I^2^: 99.94%, *p* < 0.0001). The funnel plot revealed no risk of publication bias, and further confirmation was given by Egger’s test (*p* = 0.51). Table 4 shows the forest plot of the meta-analysis, and Figure 3 shows the funnel plot for the publication bias.

## 4. Discussion

Our data show that immigrants in Europe were a high-risk group for contracting COVID-19 because of mistrust of the relevant vaccine, but indications are lacking for many nations on the European continent. Despite our decision to expand the consultation of articles published in four different European languages, we were able to gather data only from nine European nations (Denmark, Norway, Finland, France, Germany, Italy, Sweden, Switzerland, and Turkey). However, our review covers data on a relevant number of immigrants (a total of 2,009,820) living on the European continent during the COVID-19 epidemic. In particular, our systematic review reported immigrants’ willingness to vaccinate or their hesitation/refusal, indicating when possible what barriers hindered vaccine uptake and what factors were found to be determinants of under-immunization in immigrant populations. The results show that vaccination efforts in European countries need to be strengthened to ensure lifelong vaccination of immigrants in line with Crawshaw et al. [10], who recently pointed out that numerous barriers hinder immigrants in the vaccination process from communication difficulties to barriers attributable to health services. However, the problem appears to be mainly cultural, distinguishing a different propensity according to sex and ethnic origin strongly conditioned by a different trust in the host country’s institutions and health care, as we will discuss below.

COVID-19 vaccine hesitancy/refusal and related factors have been partially examined by some previous reviews (among others: [34,35,36,37]). However, these studies did not specifically focus on first-generation immigrants in Europe and provided a partial picture based on vaccine hesitancy in the early phase of the COVID-19 epidemic. Our results aim, on the one hand, at a targeted study on first-generation immigrants and, on the other, at an updated analysis of the responses reported by studies in the literature during the entire pandemic period.

According to this systematic review, we analyzed which variables were found to be determinants in the decision to vaccinate against COVID-19 in the immigrant population. The first determinant variable was sex. In contrast to the findings of Gram et al. [21] (similar vaccination coverage between sexes), Bastola et al. [18] (lower vaccine uptake in males from the total sample), and Page et al. [28] (increased female willingness to be vaccinated), female immigrants were generally more hesitant and/or opposed to vaccination than male immigrants [16,19,22,25,27,29,30]. We believe that this female attitude may be influenced by a greater attachment to the culture of the country of origin, and, consequently, immigrant women have a greater fear/suspicion of accepting directions from the institution in the host country. The same role associated with motherhood may have been influential: women are often hostile to vaccinations for their children and prefer to rely on practices managed by them (feeding, nutrition, and natural living) [38]. We can also hypothesize that, in the case of family reunification immigration, women remain at home with their children and consequently achieve lower language proficiency than males, with greater difficulty in understanding within health facilities. Educational attainment, which is often lower than that of individuals of the opposite sex from the same country, may also further aggravate the situation. However, language difficulties will decrease with the length of stay in the host country [39]. It has also been hypothesized that a different attitude toward their bodies and the socialization process may account for their different disposition toward vaccination [40,41]. In some cases, caution may have led several women to refuse the vaccine out of concern that it was unsafe not only for themselves but also for the baby they were carrying. In general, such an attitude is common among all pregnant women, regardless of their origin, even though there was a finding of no adverse outcomes [42]. Confirming this, studies recently conducted in the UK showed that the hesitation toward the COVID-19 vaccine of a general sample of pregnant women was caused by fear of the risks to them or their children of both COVID-19 disease and the potential side effects of vaccination [43,44]. In this respect, the need for adequate communication and support from health professionals so that the importance of vaccination during pregnancy is understood by pregnant women must be emphasized [45].

Another important factor in vaccine willingness is age. According to the results reported in this systematic review, lower vaccine uptake is generally associated with younger ages while willingness increases with increasing age [16,17,18,21,22,28,30], except for two studies that show different trends (decreased willingness to vaccinate at ages >50 years old [27] and in age groups 50–59 and >70 [29]). In general, the influence of the age factor on vaccination may have been conditioned by awareness of the serious outcomes of the disease for the elderly [46]. At the same time, the higher incidence of comorbidities in the elderly may have reinforced the decision to vaccinate following key health recommendations [47]. Indeed, during the different epidemic waves, there has been an increase in the demand for anti-COVID-19 vaccine doses, especially in fragile populations such as the elders, cancer patients, and the immunocompromised [48].

The willingness to vaccinate seems to be conditioned by the immigrant’s country of origin. In particular, European immigrants would be less willing than non-European immigrants [22,26,30]. The more pronounced hesitancy in vaccination in immigrant populations from Eastern European countries, found in several studies, has cultural roots and depends on the lower trust these populations have in the governments and health services of their countries of origin. In particular, Eastern European immigrants displayed low levels of trust regarding the government’s pandemic management [26]. Among non-European immigrants, those of Asian origin were found to be the most willing to vaccinate [23], while Africans showed a high degree of hesitation [25]. In contrast to the general findings, Diaz et al. [19] found no differences in willingness to vaccinate attributable to the country of origin in the immigrants examined. In general, given the strong differences among immigrant groups from different countries in their willingness to vaccinate even in a condition of extreme emergency such as the one that resulted from the COVID-19 outbreak, preventive interventions should be oriented and tailored toward the cultural specificities of different communities. The use of even the native language in communication aimed at a given community and the involvement of health professionals in providing clear explanations about vaccination and possible side effects can generate trust and reduce inequalities in access to vaccination systems of the immigrant population. The importance of robust scientific messaging to tackle particular issues of community trust was found to be central to the distribution of the COVID-19 vaccine [49].

Finally, lower willingness to vaccinate was found to be associated with a lower level of education and income, mistrust in the authorities and health-care system, weak health literacy, absence of chronic comorbidities, poor perception of the benefits associated with vaccination, a short period of residence in the host country, poor language skills, discrimination experiences, and mental distress [17,18,19,21,22,23,24,25,26,27,28,30]. In the general population, the short period between the production of the new vaccines and the authorization to administer them after a short testing period was certainly also an additional factor that may have induced hesitation in vaccination [50]. Regarding the issue of health literacy, we would like to emphasize that, within the framework of the Council of Europe, the Steering Committee on Human Rights in Biomedicine and Health [51] has prepared a health literacy guide for equitable access to health care to empower all people, including those in vulnerable situations such as immigrants, to effectively access health services and make appropriate decisions about their health. The Guide, available in six European languages, can be freely downloaded from the website (CDBIO) [51].

Looking more broadly at the quality of studies reviewed in the systematic review using NOS, it is necessary to point out that 31% of them show a score placing them in the low-quality category with a consequent high risk of bias. This was mainly due to the lack of control for possible confounding factors, but also to unsatisfactory and/or unjustified sample sizes and unassessed or unsatisfactory response rate according to the adapted scale from Hillen et al. [14]. Anyway, it is noteworthy that most studies (69%) reported a total NOS score falling into a moderate (25%)- or high (44%)-quality category with a consequent reduced risk of bias.

After the systematic review and based on the literature included in the present review, we can reach meta-analytic conclusions on vaccination acceptance in immigrants and compare them to the population of the host country by combining standardized effects sizes among different studies, as follows:

*Vaccination uptake in immigrants*. Based on nine samples from eight studies, the estimated pooled vaccination uptake for COVID-19 by immigrants in Europe was about 71%. The rates found in the different studies selected for this review are similar to this pooled estimate except in the studies of Bajos et al. [17] (in racially minoritized immigrants from the Maghreb, Turkey, Asia, and sub-Saharan African countries in France) and Bastola et al. [18], where vaccination uptake was considerably lower (59.1% and 61.4%, respectively). The most likely explanation for the significant heterogeneity among the different studies lies in the different ethnic composition of the immigrant samples examined.

*Vaccination uptake in immigrant vs. host populations*. The meta-analysis in this case was based on seven samples from six studies. The difference in vaccination uptake was to the advantage of the host population (an estimated pooled difference of 13%): in all studies, immigrants had a lower percentage of vaccination than the host population except in the non-racially minoritized sample of Bajos et al. [17]. The significant heterogeneity among studies again probably depends on the different ethnic composition of the immigrant samples, socio-demographic disparities in access to information and COVID-19 vaccines, and the different responses to vaccination by non-immigrants according to the European country under consideration and its rules.

Taking into consideration the limitations of the studies included in this review, several of them do not report data on vaccination in the host country population, thereby reducing the possibility of including the data on immigrant vaccination adherence in a more general picture. In the course of the pandemic, moreover, different European governments showed significantly different strategies to the extent that, at certain times, they accepted the higher mortality rate that a temporarily more liberal policy entailed, as happened first in Sweden and later in the UK [52]. However, no reference was generally made in the studies considered to the policy implemented by the host country in the pandemic wave under consideration. Some studies were limited to assaying immigrants’ intention to vaccinate, while others only considered the vaccinated (with only 1 dose up to 3 doses). Moreover, there is a lack of information on whether immigrants who were reported as unvaccinated had already been vaccinated in their country of origin. Concerning this, there may have been an underestimate, especially in some countries such as Norway, because a fee is charged to enter data on vaccinations given abroad into immunization registries. Undocumented immigrants have generally not been considered in studies in the literature (except for the study of Page et al. [28] and Longchamps et al. [25]). Some factors, such as religion or media influence, have seldom been considered in the process of joining or not joining the vaccination. Some studies covered only the first waves of the epidemic, so it is not possible to assess whether vaccination rates have subsequently increased in that country. Indeed, it is known that the willingness to vaccinate against COVID-19 has changed over time as large-scale immunization programs encouraged even the most hesitant to get vaccinated [28]. Sometimes, the use of online platforms in the language of the host country may have ended up selecting only the most cultured sample. Almost all studies used a cross-sectional design preventing inferences about causality.

### Strengths and Limitations

We conducted this systematic review and meta-analysis to overview the literature on the willingness of immigrants in Europe to vaccinate and the factors that possibly influenced this decision during the recent COVID-19 outbreak, to draw lessons for the future. The main strength of this systematic review concerns compliance with the PRISMA statement and the involvement of meta-analysis. Another important point for general knowledge of the phenomenon concerns the decision to analyze all first-generation immigrants including irregular immigrants. It should be noted, however, that the majority of the selected studies refer to in-country residents or do not specify the status of the immigrant. In addition, to reduce heterogeneity among studies and rely on definite data, we did not consider those based on vaccination intention in the meta-analysis but applied this analysis only to studies that reported certain data on vaccination or relative refusal.

In addition to the strengths, some weaknesses are to be added to those peculiar to individual studies that we have already listed, as follows: (i) Different survey instruments were used to assess hesitancy/refusal to vaccinate with consequent possible influences on the data collected in the systematic review and resulting in a reduction in the number of studies that can be used for meta-analysis; (ii) The studies were conducted at different times in the epidemic and refer to different waves of COVID-19 (some were carried out before the vaccines were even produced). Consequently, we cannot exclude that the data on hesitancy to vaccination were affected by the particular context of the different waves of the COVID-19 epidemic during which the surveys were conducted; (iii) The different policies adopted in vaccine communication and administration of the different European countries examined may have influenced immigrants’ decision to vaccinate; (iv) Because of the few European studies that reported data on COVID-19 vaccination acceptance in immigrants or the even fewer studies that compared them with the host country population, it was only possible to enter data into the multivariate analyses respectively from 8 and 6 studies, resulting in reduced statistical power.

## 5. Conclusions

The review conducted allowed us to find that some common shortcomings in European countries need to be addressed to improve access to vaccinations for immigrants. In particular, the lack of trust in the government and the local healthcare system deserves to be tackled taking into account the immigrants’ cultural differences, with particular regard to Eastern European migrants. From this point of view, it would be relevant to longitudinally assess the relationships between willingness to vaccinate and immigrant acculturation (i.e., the process of adoption, acquisition, and adjusting to the new cultural environment). Data on policies to include and facilitate access to the health-care system by undocumented immigrants are severely lacking in all European countries. Immigrants, especially if irregular, represent a particularly fragile segment of the population due to precarious living conditions, in unhealthy and crowded places, with poor hygiene. It is therefore necessary for public health to protect all those who live in conditions of social fragility by guaranteeing them the same health rights as the rest of the population. Ignoring this, as well as being ethically reprehensible, can lead to a general deterioration in the health of the entire population, especially in the event of epidemics. European governments must therefore work to ensure equal availability of COVID-19 vaccines and other health-saving ones for all immigrants.

Another important aspect that could be addressed in future studies concerns the analysis of second-generation immigrants as this could clarify some more specific effects of the acculturation process.

## Figures and Tables

**Table 1:** Summary of the quantitative studies included in alphabetical order.

Authors (Year) -Study Design-	Host Country	Country of Origin	Participant Groups (Sex, N, Education, Income, Comorbidities)	Age (Years)	Year and Method of Data Collection	Infection and/or Prevalence of COVID-19 Vaccination Willingness and Hesitancy	Main Factors of Refusal or Hesitancy to COVID-19 Vaccination
Aysit et al. (2023) [16] -cross-sectional-	Turkey	Syria	In the target group of 911 Syrians Under Temporary Protection in Istanbul, 598 questionnaires were distributed, including 571 people. 80.7% women. 26.6% high school level. 24.7% with chronic diseases. 45% with chronic diseases in the family. Mean duration of stay in Turkey: 6.14 ± 2.22 years.	≥18 years 31.92 ± 6.14	February, March, and May 2022 Questionnaires translated into Arabic	**Vaccinated:** 56.4% of males and 42.3% of females; 54.4% aged ≥35 years; 50% high school and above; 68.6% with regular job; 54.6% with chronic disease and 54.1 with a chronic disease in the family. *The local population has a rate of at least one dose of vaccine above 90%* vs. *45% of immigrants.*	The study shows by logistic regression that the variables associated with COVID-19 vaccination status are male sex, older age, middle/upper economic status, and the presence of chronic diseases in the family. In addition, vaccination was more frequent in individuals with high levels of health literacy.
Bajos et al. (2022) [17] -random population-based cohort survey-	France	Partially specified for people native to French Overseas Departments (FOD) (Martinique, Guadeloupe, Reunion Island, Guyana, and Mayotte) and racially minoritized groups (immigrants or descendants of immigrants from the Maghreb, Turkey, Asia, and sub-Saharan African countries)	80,971 persons living in metropolitan France. Among them there were native to FOD, racially minoritized first-generation and second-generation immigrants, non-racially minoritized first-generation and second-generation immigrants. All others constitute the mainstream population.	≥18 years	July 2021 Computer-assisted web interviews (CAWI) or computer-assisted telephone interviews (CATI)	**Vaccinated with at least 1 dose**: 74.5% mainstream pop.; 56.2% born in FOD; racially minoritized groups: 52.5% (2nd generation) and 59.1% (1st gen); other immigrants: 75.6% (2nd gen) and 76.3% (1st gen). **Refusal to vaccinate**: 7.8% mainstream pop.; 14.2% born in FOD; racially minoritized groups: 12.8% (2nd generation) and 7.4% (1st gen); other immigrants: 7.1% (2nd gen) and 7.0% (1st gen). **No trust at all in government**: 17.5% mainstream pop.; 21.3% born in FOD; racially minoritized groups: 20.2% (2nd generation) and 11.8% (1st gen); other immigrants: 16.3% (2nd gen) and 12.1% (1st gen). **No trust at all in scientists**: 3.6% mainstream pop.; 8.9% born in FOD; racially minoritized groups: 6.8% (2nd gen) and 4.4% (1st gen); other immigrants: 3.2% (2nd gen) and 2.9% (1st gen).	The unvaccinated were found to be younger, less educated, lower income, and often from racially minoritized groups. The factors most associated with vaccination refusal were a lack of trust in the government and scientists to contain the spread of the epidemic.
Bastola et al. (2023) [18] -cross-sectional-	Finland	Russia, Estonia, Europe (excl. Russia, Estonia)/North America/Oceania, Middle East/North Africa, Africa (excl. North Africa), Southeast Asia, Asia (excl. Southeast Asia)/Latin America	13,223 immigrants in Finland (48% females). FinMonik (based on the Survey on Wellbeing among Foreign-Born Population) sample: 89.3% lived in urban centers. 82.6% were aged ≥18 years at migration. Length of stay in Finland was ≥12 years in 51.9% of the sample. Among MigCOVID (based on the Impact of Coronavirus Epidemic on Wellbeing among Foreign-born Population) subsample (3668 immigrants): secondary level of education (46.3%); full-time or part-time workers for 64.5%; beginning or intermediate knowledge of the language in 64.2%; no psychological distress in 80.3%; no perceived discrimination 85%; perception of fairly good/good health in 71.8%.	20–66 years	October 2020–February 2021 Registers, electronic or paper-based questionnaires in 18 languages, and multilingual telephone interviews	The highest incidence of infection was in immigrants from Africa (19.4%), and the lowest was in immigrants from Southeast Asia (5.1%). 61.4% of immigrants completed vaccine **uptake**: the complete vaccination uptake ranged from 85.0% (Southeast Asians) to 41.0% (Estonians).	In the FinMonik sample, the male sex, younger age, migration age <18 years, and shorter length of residence were associated with lower COVID-19 vaccine uptake. In the MigCovid subsample, younger age, being economically inactive, poorer language skills, experiences of discrimination, and psychological distress were associated with lower vaccine uptake.
Diaz et al. (2022) [19] -cross-sectional-	Norway	Sweden, Pakistan, Philippines, Poland, Somalia	1284 immigrant residents in 6 parishes in Oslo (50.9% females). Education: no University: 40.4%. *3596 non-immigrants in the same parishes in Oslo (60.5% females).* *Education: no University: 37.8%.*	≥18 years	16–24 June 2021 web-based survey	**Vaccine offer**: 68.1% of immigrants vs. 81.1% of non-immigrants **Vaccine uptake** (% of those offered): 79.9% in immigrants vs. 91.1% in non-immigrants Confirmed case of COVID-19: 8.4% in immigrants vs. 4.0 in non-immigrants	There are disparities between non-immigrants and immigrants in actual vaccine uptake. The vaccine offer was lower in immigrants than in non-immigrants. Females were more likely than males to receive the vaccine, while individuals with some university education were less likely. No differences were found by language spoken at home, or by country of origin. While length of residence was found to be an important explanatory variant, immigrants who had lived in Norway for less than 15 years were less likely to refer to the vaccine offer.
Führer et al. (2022) [20] -cross-sectional-	Germany	Germany, Turkey, Syria, Venezuela, Iran, etc.	204 immigrants residing in Germany (57.1% females). Education: more than 10 years in 80.2% of immigrants. Median duration of stay in Germany: 6.5 years	≥18 years Mean age: 37 years	September 2021–January 2022 German online questionnaire translated into 5 more languages	**Acceptance** Importance of COVID-19 vaccine: yes in 68.6%, no in 18.9%, undecided in 7.3%. Fear of side effects: 55%; no fear: 26.7%. Safe vaccination: 31%; no safe vaccination: 47.9%. Non-dangerousness of COVID-19: 42.7%; dangerousness:37%. Finally, 26% believe that nature should take its course, 52% believe in natural remedies, and 26% are afraid of syringes. **Vaccination status** 80% (n = 154) had already received at least one dose of COVID-19 vaccine; 2% (n = 4) had not been vaccinated but already had an appointment for vaccination; 17% (n = 33) had no appointment.	The overall vaccination rate of immigrants was comparable to that of the general population. The high vaccination rate found in this study may depend on the high level of education and safe residence status of the respondents.
Gram et al. (2023) [21] -nationwide register-based cohort study-	Denmark (5 geographical regions)	Immigrants of Western descent (born abroad, without any parent Danish citizen or born in Denmark), descendants of Western immigrants (born in Denmark, without any parent Danish citizen or born in Denmark), immigrants of non-Western descent (born abroad, without any parent Danish citizen or born in Denmark), descendants of non-Western immigrants (born in Denmark, without any parent Danish citizen or born in Denmark).	Overall, 5,164,558 individuals (50.5% females). By immigrant status: 341,830 immigrants of Western descent, 15,300 descendants of Western immigrants, 367,200 immigrants of non-Western.descent, 108,475 descendants of non-Western immigrants *4,330,606 Danish (born in Denmark or abroad with at least one parent who is both a Danish citizen and born in Denmark)*.	≥12 yrs Overall mean age: 47 ± 20.7 yrs	From 27 December 2020 to 20 October 2021. Danish registries on infections and vaccinations	**Vaccination coverage** ranged between 85.5% and 88.7% across the 5 geographical regions. 87.1% received at least the first dose of COVID-19 vaccine. The coverage was similar between sexes, and lower in: -younger group (12–15 yrs) (70%); -no Danish individuals especially in descendants of non-Western immigrants (49.2%); -individuals without chronic diseases (86.0%); -individuals with an income <33,605 € (85.0%); -individuals with upper secondary school (85%) or primary school (83.9%) -individuals with a previous SARS-CoV-2 infection (79.5%); -individuals never PCR-tested (69.6%).	There was a high COVID-19 vaccine uptake in Denmark, but large socio-demographic differences in vaccine uptake have been identified that particularly affected younger age groups.
Holz et al. (2022) [22] -cross-sectional data collected via a third-party online access panel provider-	Germany	Countries of origin: 63.3% Europe; 21% non-Europe; 15.6% other	477 first-generation immigrants residing in Germany (53.7% females). Education: 63.6% secondary degree. Mean years since migration: 22.6 ± 16.38. *532 native Germans without migration background (47.1% females).* *Education: 56.2% secondary degree.*	18–65 years Immigrants mean: 41.56 ± 12.71 years *Non-immigrant Germans mean: 44.13 ± 13.67 yrs.*	15 March 2021–25 March 2021 Participants randomly drawn from the database of the panel provider and incentivized by earning 50 Euro-Cents per 10 min interview time	**Vaccination intention** % Definitely: 34.9% of immigrants vs 51.4% in native Germans. Not at all: 21.1% of immigrants vs. 14.2% of native Germans.	An increase in positive antecedents such as religiosity, which positively influence general attitudes such as fears of infection and intention to vaccinate were found in immigrants. However, a significant direct negative association with vaccination was also found in immigrants. Political trust and health consciousness have increased over the years since migration. European immigrants have less political trust, fear of personal contagion, and lower intention to vaccinate than non-European immigrants.
Kraft et al. (2021) [23] -cohort study-	Norway Different lengths of residence (<6 years, 6–10, 11–15, 16+ years of residence) were considered	Norway, Vietnam, Sri Lanka, Thailand, Denmark, Philippines, India, UK, Sweden, Iran, Iceland, Pakistan, Brazil, Finland, Netherlands, USA, Afghanistan, Bosnia-Herzegovina, Chile, China, Kosovo, Turkey, Germany, Ethiopia, Iraq, Syria, Morocco, Spain, Eritrea, France, Serbia, Ukraine, Somalia, Croatia, Russia, Lithuania, Poland, Bulgaria, Romania, Latvia, etc.	746,062 Norway residents with a migratory background including 689,540 foreign-born, and 57,153 Norwegian-born with foreign-born parents (48% females). Higher education: foreign-born 32.00%; Norwegian-born with foreign-born parents 39.18%. Income: foreign-born: NOK 299898; Norwegian-born with foreign-born parents: NOK 304288. *Reference group: 3,518,308 Norwegian-born with Norwegian-born parents (50% of females)* *Higher education:* *36.30%.* *Income: NOK 402683*	≥18 years Foreign-born: 44 ± 14 yrs Norwegian-born with foreign-born parents: 29 ± 12 yrs *Norwegian-born: 50 ± 19 yrs.*	At least 1 dose from 8 December 2020, to 20 October 2021. National Register of COVID-19 vaccinations	**Vaccinated** with at least 1 dose -Foreign-born: 73% -Norwegian-born with foreign-born parents: 82% *(Norwegian-born: 93%)*	COVID-19 vaccination coverage is lower in immigrant groups in Norway than in Norwegian-born persons. Based on different country backgrounds, vaccination coverage is high and hovers around 90% in immigrants from countries such as Vietnam (93%), Sri Lanka (91%), Thailand (91%), Denmark (89%), Philippines (89%), India (88%), the UK (88%), Sweden (88%), and Iran (87%). Relatively low coverage is observed in immigrants from countries such as Latvia (44%), Bulgaria (45%), Romania (45%), Poland (46%), and Lithuania (47%). There are also differences among immigrant groups partly attributable to income and education.
Lajunen, Wróbel (2022) [24] -cross-sectional-	Norway	Poland	150 Polish first-generation immigrants in Norway (47.7% females). *256 Poles living in Poland (52.3% females).* *264 Norwegians living in Norway (63.2% females).*	≥18 years Immigrants mean: 42.2 ± 11.8 yrs *Poles living in Poland mean: 42.9* ± *11.8 yrs* *Norwegians mean: 38.9* ± *18.4 yrs*	March–May 2021. Internet-based anonymous survey	**Attitude to the COVID-19 vaccine** was assessed using a scale constructed as the average of answers to eight items about the COVID-19 vaccine recorded with a five-point Likert scale from “totally disagree” (1) to “totally agree” (5): Poles in Norway: 3.08 ± 1.29 *Poles in Poland 3.29 ± 1.14* *Norwegians in Norway 4.31 ± 0.55*	The attitudes of Polish immigrants in Norway toward COVID-19 vaccination were found to be similar to Polish immigrants living in Poland. Confidence in the competence and values of the Norwegian health-care system among Polish immigrants was significantly lower than among the Norwegian population without immigrant background. This mistrust results in lower immunization rates among Polish immigrants, with a consequent possible increase in unnecessary suffering and health inequalities.
Longchamps et al. (2021) [25] -cross-sectional ECHO study-	France (Paris, Lyon, Strasbourg)	Europe (15.6%) Africa (59.5%) Eastern Mediterranean (21.5%) Other (3.4%)	N = 235 residents in 18 homeless shelters (66.3% males; 62.9% secondary school or less; 70.5% not employed 60.8% no legal residence in France). 27.9% Depression 25.5% chronic disease 76.4% trust in official information on COVID-19	19.3% of 18–24 y; 41.8% of 25–34 y; 25.9% of 35–49 y; 13% of ≥50 y	2 May–28 June 2020 (before anti-COVID-19 vaccines were approved for use); Interview in person or by telephone Questionnaire administered in French, English, or the participant’s language (25% of the questionnaires were completed with the assistance of a trained translator contacted by telephone)	**Vaccine hesitancy** 40.9% (71.2% No; 28.8% I don’t know) **By sex:** Male: 32.5% Female: 55.5% **By country of birth:** Europe: 52.7% Africa: 43.6% Eastern M. 30.0% Other: 12.5% **By household composition**: Living alone: 36.9% Living with a partner: 56.1% **By administrative status:** Legal residence: 48.9% No legal residence: 35.9% **By health literacy:** Low: 50.9% Intermediate/high: 31.3% *Findings are in line with observations from general population surveys*	Determinants of vaccine hesitancy were sex, household composition, administrative status, and health literacy: being female (OR 2.55), living with a partner (OR 2.48), without legal residence (OR 0.51), and with low health literacy (OR 0.38) were associated with vaccine hesitancy. Women are more likely to express vaccine hesitancy than men. Health care is not always adapted to immigrants’ needs, resulting in inadequate or negative experiences. Low health literacy means limited capacity to seek and evaluate health information All messages on measures that can prevent COVID-19 should be adapted to be easily understood by all.
Madar et al. (2022) [26] -cross-sectional. It is part of the project Inncovid-	Norway	Poland, Spain, India, Somalia, Arabic language area	529 immigrants living in Norway (47.6% females). Ethnicity: 33 Somali, 137 Arabic, 72 Tamil, 113 Spanish, 174 Polish. Years in Norway: 67.3% over 5 years. Health status: 60.7% excellent/very good	≥18 years 81% between 26 and 55 years	Between 25 May and 1 July of 2020. Online survey. A questionnaire translated into five different languages (Polish, Arabic, Somali, Tamil and Spanish) with a completion time of around 15 min	**COVID-19 infection** -No (documented or presumed) in 96.5% of immigrants. -Yes (documented or presumed) in 3.7% of immigrants **Information from health authorities:** -Yes in 82% of immigrants **Inaccuracy of information from social media:** -Agree in 78.6% of immigrants	Immigrants in Norway believe they have received adequate information about COVID-19 and have high compliance with preventive measures, although many variations between groups were found. In particular, Poles reported the lowest levels of trust while Tamil- and Arabic-speaking respondents reported high levels of trust in the health-care system. The majority of immigrants agreed that by following the recommendations, they could avoid getting sick, but more skepticism was found among the Spanish and Polish.
Maifredi et al. (2022) [27] -cohort study-	Italy (Brescia)	Foreigners (inhabitants with non-Italian citizenship at enrollment)	134,492 foreigners (51.4% females). Comorbidity: none (76.9%); 1 (15.6%); 2–3 (7.1%); >3 (1.40%). *869,718 Italians (50.9% females).* *Comorbidity:* *none (53.1%);* *1 (20.1%);* *2–3 (18.3%);* *>3 (8.0%).*	>18 years Foreigners median: 40.6 yrs (31.5–50.8) *Italians* *median: 53.7 yrs* *(39.6–68.5)*	30 September–31 December 2021. Brescia Local Health Agency Database	**Unvaccinated** (**%**) On 31 December 2021: -25.3% of foreigners *-8.7% of Italians*	Diagnosis of SARS-CoV-2 infection may be less attainable for foreigners due to misinformation, language barriers, and lack of trust in traditional medicine. This would explain the fact that only severe cases reached hospitals. The probability of undergoing COVID-19 vaccination increased with the male gender and the number of comorbidities among foreigners, while, among Italians, also age (50–69, or >70) contributed. Hesitancy to vaccinate against COVID-19 was higher among foreigners than among Italians. The likelihood of not being vaccinated among foreigners was significantly higher in women, people without chronic comorbidities, and people aged >50 years.
Page et al. (2022) [28] -multicentric cross-sectional survey-	Switzerland (Geneva), Italy (Milan), France (Paris)	Africa, the Americas, the Eastern Mediterranean, Europe, Asia, Western Pacific.	670 undocumented immigrants: Geneva N = 441 (63.4% females); Milan N = 126 (67.2% females); Paris N = 103 (30.1% females).	>16 yrs Mean Age (yrs): Geneva 39 ± 17; Milan 41 ± 20; Paris 35 ± 16	February–May 2021. Anonymous structured questionnaire translated into 10 languages available at health facilities.	**Willingness to vaccinate** -52% in Milan vs. 82% in Italy (December 2020) -14.6% in Paris vs. 71.8 in France (June 2020). -39% in Geneva	The vast majority of participants had perceived that COVID-19 vaccination was accessible to all, while the remainder predominantly thought they could not access it due to lack of health insurance. The low demand for vaccination was related to age, comorbidity, and views on vaccination. Women were more likely to be vaccinated than men. Social media (Milan) and community networks (Paris) were found to be negatively associated with demand. Hospitals were the preferred place for vaccination
Russo et al. (2021) [29] -cohort study-	Italy (Metropolitan Area of Milan: residents in Milan and Lodi)	Not reported	2,981,997 persons (52.1% females). 414,920 persons with foreign nationality.	≥19 years	Between 1 January and 30 September 2021. A new regional registry (NAR) of caregivers integrated with information from the permanent geo-referencing system.	**Vaccinated with at least 1 dose**: 73.2% immigrants 86.2% Italians -51.52% males -48.48% females	Women are less likely to get vaccinated than men, as are the age groups 50–59 years and 70+ years. In addition, residents of more deprived areas were found to be less likely to be vaccinated than those living in more affluent areas. Italian citizens were found to be more likely to vaccinate than foreigners. This willingness increased as the number of chronic conditions they had increased. Uptake of the vaccination campaign is influenced by the socio-demographic characteristics of the population and is a determining factor in the prevention of hospitalizations.
Svallfors et al. (2023) [30] -cross-sectional study-	Sweden	Middle East and North Africa (53%); Sub-Saharan Africa, Eastern Europe; Western Europe, North America, Australia or New Zealand; South America; Asia.	2612 first-generation immigrants (49% females). Secondary level of education: 36%. Cohabiting or married: 63%. Place of residence: city (41%) or big city (36%). 18% had somewhat low or very low trust in Swedish authorities.	≥16 yrs	April–May and August–September 2021. computer-assisted per- sonal interviews and web interviews. The survey was developed in Swedish and translated into 7 additional languages.	**Vaccine hesitancy:** 77% were vaccinated or intended to be vaccinated, considering it very important for themselves and others (58%). 21% had already had the infection. Of the 23% who were hesitant, 5% said they would not vaccinate, 7% probably not, 4% did not know, and 7% did not want to answer. 11% do not think vaccination is important for their health and 7% for others’ health. When this study started, 81% of Swedes over the age of 16 were fully vaccinated vs. 68% of those born abroad (August 2021).	Determinants of COVID-19 vaccination hesitancy were found to be: young age, arrival in Sweden during the 2015 migration wave, Eastern European origin, female gender, lower education level, lack of trust in authorities, and lower perception of vaccination benefits.
Vinjerui et al. (2022) [31] -cross-sectional-	Norway	Immigrants born in 22 EU countries: Austria, Belgium, Bulgaria, Denmark, Estonia, Finland, France, Iceland, Italy, Croatia, Latvia, Lithuania, the Netherlands, Poland, Portugal, Romania, Slovakia, Spain, Sweden, Czech Republic, Germany, Hungary.	276,506 residents born abroad (46% females). Period of residence <6 yrs: 17%. Median income: 344,282 NOK *3,575,107 Norwegians (50% females). Median income: 401,387 NOK*	≥18 years Immigrants mean: 43.14 ± 14 yrs *Non-migrant Norwegians mean: 50 ± 19 yrs*	By 30 September 2021 National Register of COVID-19 vaccinations	Vaccination **coverage** ranged from 24.3% (in Bulgaria) to 98.1% (in Portugal) in European countries, from 44.0% to 89.2% among immigrants of European descent in Norway in September 2021. Bulgarian immigrants showed the lowest COVID-19 vaccination coverage compared to other immigrants in Norway. Bulgaria was also the birth country in Europe with the lowest percentage of vaccinated people.	A covariation was found between the vaccination coverage of immigrant groups from Europe and the vaccination rate in their countries of birth. Vaccination coverage was higher in immigrants with a long period of residence in Norway than in those with a short period of residence, but there was no significant difference in the covariation with the country of birth for people who lived in Norway for a long or short period of residence.

Note: Data on the reference population are in italics.

**Table 2:** Quality assessment of included studies by Newcastle–Ottawa Scale (NOS) with higher scores indicating better quality research. The relative range is shown for each item.

References in Alphabetical Order	Clarity of Stated Aim *(0–2)*	Sample Selection				Comparability		Outcome		NOS Score *(0–16)*
		Sample Representativeness *(0–2)*	Sample Size *(0–2)*	Non-Respondents (0–2) *	Ascertainment of the Exposure *(0–2)*	Control of Confounding Factors *(0–1)*	Comparability of Participants *(0–1)*	Assessment of the Outcome *(0–2)*	Statistical Tests *(0–2)*	
Aysit et al. (2023) [16]	2	1	2	1	2	0	0	2	2	12
Bajos et al. (2022) [17]	2	2	2	1	1	1	1	1	2	13
Bastola et al. (2023) [18]	2	2	2	1	2	1	0	2	2	14
Diaz et al. (2022) [19]	2	0	1	1	1	1	1	1	2	10
Führer et al. (2022) [20]	2	0	0	0	2	0	0	1	2	7
Gram et al. (2023) [21]	1	2	2	2	2	1	1	2	2	15
Holz et al. (2022) [22]	2	1	1	0	1	1	1	1	2	10
Kraft at al. (2021) [23]	1	2	2	2	2	1	1	2	2	15
Lajunen, Wróbel (2022) [24]	2	0	0	0	1	0	1	1	1	6
Longchamps et al. (2021) [25]	1	1	1	0	1	0	0	1	2	7
Madar et al. (2022) [26]	2	1	1	0	1	0	0	1	1	7
Maifredi et al. (2022) [27]	1	2	2	2	2	1	1	2	2	15
Page et al. (2022) [28]	1	0	1	2	1	1	0	1	2	9
Russo et al. (2021) [29]	2	2	2	2	2	1	1	2	1	15
Svallfors et al. (2023) [30]	1	1	1	2	1	0	0	1	1	8
Vinjerui et al. (2022) [31]	1	2	2	2	2	0	1	2	2	14

* As carried out previously [32,33], we assigned the score 0 to the third item in the Non-respondents category of the NOS-adapted scale [15].

**Table 3:** Studies used in the meta-analysis and the forest plot of the pooled proportions (%) of COVID-19 vaccinated immigrants.

Study or Subgroup	Sample Size	Proportion (%)	95% CI	Weight (%)	
Bajos (2022) [17] *	2799	59.09	57.25 to 60.92	10.96	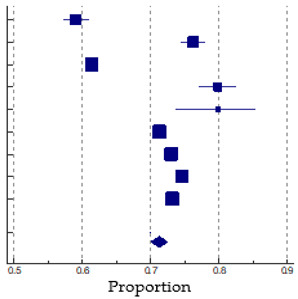
Bajos (2022) [17] **	2629	76.30	74.63 to 77.92	10.83	
Bastola (2023) [18]	13,223	61.40	60.57 to 62.23	12.97	
Diaz (2022) [19]	875	79.89	77.07 to 82.50	7.67	
Fuhrer (2022) [20]	204	79.90	73.74 to 85.17	3.15	
Gram (2023) [21]	709,030	71.30	71.20 to 71.41	13.62	
Kraft (2021) [23]	689,540	73.00	72.90 to 73.11	13.62	
Maifredi(2022) [27]	127,504	74.61	74.37 to 74.85	13.56	
Russo (2021) [29]	414,920	73.22	73.09 to 73.36	13.61	
**Total**	**1,960,724**	**71.28**	**70.02 to 72.52**	**100.00**	

Note: Bajos *: racially minoritized groups; Bajos **: non-racially minoritized groups. The squares and error bars signify the proportion and 95% confidence interval (CI) values, respectively. The diamond represents the pooled effect sizes.

**Table 4:** Studies used in the meta-analysis and forest plot of the difference in vaccination uptake in immigrant vs. host populations.

Study	Difference	95% CI	Weight (%)	
Bajos(2022) [17] *	−0.154	−0.173 to −0.136	13.97	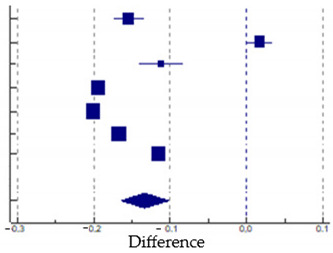
Bajos(2022) [17] **	0.018	0.001 to 0.035	14.11	
Diaz (2022) [19]	−0.112	−0.141 to −0.084	13.07	
Gram (2023) [21]	−0.194	−0.195 to −0.193	14.72	
Kraft (2021) [23]	−0.200	−0.201 to −0.199	14.72	
Maifredi (2022) [27]	−0.167	−0.169 to −0.164	14.70	
Russo (2021) [29]	−0.115	−0.116 to −0.114	14.71	
**Total**	**−0.133**	**−0.164 to −0.102**	**100.00**	

Note: Bajos *: racially minoritized groups; Bajos **: non-racially minoritized groups. The squares and error bars signify the difference values and 95 % confidence interval (CI) values, respectively. The diamond represents the pooled effect sizes.

## Data Availability

Not applicable.

## Supplementary Materials

The following supporting information can be downloaded at: https://www.mdpi.com/article/10.3390/vaccines12040350/s1, Table S1: Prisma 2020 for Abstracts Checklist; Table S2: Prisma 2020 Checklist.
